# Fucosyltransferase 3 and 8 promote the metastatic capacity of cancer stem-like cells via CD15s and E-cadherin in esophageal cancer

**DOI:** 10.22038/IJBMS.2024.74726.16228

**Published:** 2024

**Authors:** Aliakbar Rostami Abookheili, Jahanbakhsh Asadi, Ayyoob Khosravi, Ali Gorji

**Affiliations:** 1 Department of Molecular Medicine, Faculty of Advanced Medical Technologies, Golestan University of Medical Sciences, Gorgan, Iran; 2 Shefa Neuroscience Research Center, Khatam Al-anbia Hospital, Tehran, Iran; 3 Metabolic Disorders Research Center, Golestan University of Medical Sciences, Gorgan, Iran; 4 Stem Cell Research Center, Golestan University of Medical Sciences, Gorgan, Iran; 5 Epilepsy Research Center, Westfälische Wilhelms-Universität Münster, Münster, Germany

**Keywords:** 2F-PerAcFuc, E-cadherin, Esophageal cancer stem – cells, Fucosyltransferase 3 and 8, Sialyl lewis X

## Abstract

**Objective(s):**

Esophageal cancer stem cells (ECSCs) have been identified as the subset of cells within esophageal squamous cell carcinoma that possess tumorigenic, invasive, and metastatic properties. One important aspect of cancer metastasis is the binding of sialyl-Lewis X (CD15s) with E- or P-selectin, which facilitates the adhesion and migration of cancer cells to distant sites. This study was conducted to investigate the impact of fucosylation processes on the metastatic behavior of ECSCs.

**Materials and Methods:**

The esophageal cancer cell line (KYSE-30) was cultured and divided into control and 2F-peracetyl fucose (2F-PerAcFuc) treated groups. Spheres were harvested from these cultures. Cell invasion assay and qPCR were conducted to examine migration and marker expression in both groups. Cancer cell line-derived xenografts were established in nude mice to validate findings *in vivo*.

**Results:**

Our results initially indicated that the addition of 2F-PerAcFuc, an inhibitor of fucosylation, resulted in the down-regulation of the Fut3/CD15s pathway in both cancer stem-like cells and the xenograft model. Measurements of subcutaneous xenograft tumor volume revealed a significant decrease in tumor size among nude mice after treatment with 2F-PerAcFuc. Additionally, a reduction in Fut8/E-cadherin levels was observed in the xenograft model of nude mice. Furthermore, the administration of 2F-PerAcFuc lowered the levels of fucosylated glycoconjugates in nude mice.

**Conclusion:**

Our data suggest that inhibition of fucosyltransferase 3 and 8 can reduce the metastatic capacity of cancer stem-like cells by down-regulating CD15s and E-cadherin in a mouse model of esophageal cancer.

## Introduction

The failure of current molecular cancer therapy is primarily due to tumor heterogeneity (1-3). According to the cancer stem cells (CSCs) theory, a tiny subset of cancer cells with stem cell qualities can cause tumor recurrence, promote tumor progression, resistance to drug treatments, and cause metastasis (4-6). Metastasis is a multi-step process that results in the formation of secondary tumors in distant locations from the primary tumor. It is the main cause of mortality in cancer patients and is associated with poor prognosis and treatment outcomes (7). Many metastatic tumors have been shown to exhibit aberrant glycosylation patterns. Glycosylation, the most common post-translational modification (PTM) in proteins and lipids (8], plays a crucial role in the regulation of various biological processes (9). Fucosylation, one of the most prevalent glycosylation modifications, has been found to contribute to malignant transformation processes, including invasion, metastasis, angiogenesis, and immune evasion (10-12). Increased fucosylation is also more common in CSCs (13). Fucosyltransferase (FUT) enzymes are responsible for the fucosylation process (14, 15). In the human genome, there are 13 identified FUT genes. These enzymes transfer fucose sugar molecules to α1,2, α1,3/4, and α1,6 bonds on O-peptide and N-peptide sites of different glycan molecules (16). During cell development, differentiation, and malignant transformation, fucosylated glycoconjugates participate in numerous cell-cell interactions (17, 18). CD15s, also known as sialyl-Lewis X (SLeX), are a type of fucosylated glycoconjugate expressed on the cell surface glycolipids. The final step in the biosynthesis of SLeX involves the transfer of fucose to sialylated N-acetyllactosamine-containing glycans by functionally similar α1,3 FUTs. CD15s is expressed in neutrophils (19, 20], basophils (21), and monocytes (22), but not in eosinophils (20]. The initial molecular-level interaction between vascular endothelial cells and leukocytes is mediated by the interactions between E-selectin on endothelial cells and specific oligosaccharides, particularly SLeX expressed on the cell surface of leukocytes (23). This interaction has been shown to facilitate the binding of metastatic cancer cells to endothelial cells (24), leading to extravasation of circulating cancer cells (25). Studies have identified cellular plasticity mediated by epithelial-mesenchymal and mesenchymal-epithelial transitions (EMT and MET, respectively) as a key molecular theme in metastasis initiation (26, 27). Primary epithelial tumor cells undergo EMT, acquiring migratory and invasive properties to invade distant areas, and then undergo MET to establish a metastatic epithelial lesion (28-30). E-cadherin, a component of membrane-associated protein complexes that form adhesion junctions, is involved in cell adhesion in various cell types (31). Glycosylation of E-cadherin plays a crucial role in regulating its functions in cancer (32, 33). Reduction in α1,6 fucosylation on E-cadherin strengthens E-cadherin-mediated cell-cell adhesion, while overexpression of FUT8 in lung cancer decreases it (11). Therefore, FUT enzymes are considered promising targets for preventing cancer metastasis and for effective CSC-targeted therapy. 2F-PerAcFuc is a cell-permeable fluorinated fucose derivative that functions as a fucosyltransferase inhibitor by being absorbed and metabolically transformed into a GDP-fucose mimetic (11). Based on these considerations, we hypothesized that there is a relationship between fucosylation alteration, the EMT-MET process, and the metastatic behavior of CSCs. The present study aimed to evaluate the effect of 2F-PerAcFuc on the metastatic capacity of esophageal cancer stem-like cells through fucosyltransferase inhibition.

## Materials and Methods

***Cell culture***

KYSE-30 esophageal cancer cell line was obtained from the Iranian cell bank, Pasteur Institute, Tehran, Iran. The cells were grown in Dulbecco’s Modified Eagle Medium/F12 (DMEM/F12) with 10% fetal bovine serum (FBS), 100 units/ml of penicillin, and 100 µg/ml of streptomycin at 37 °C and 5% CO2. Every 3–5 days, the cells were transferred into new T25 culture flasks (SPL Life Science, Korea) and harvested using trypsin. The cultured cells were divided into two groups: the control group and the 2F-PerAcFuc group. The 2F-PerAcFuc group was subjected to treatment with 100 µg of compound. The compound was dissolved in dimethyl sulfoxide (DMSO) to achieve the desired concentration and ensure cell safety (0.005%). The compound-treated group was exposed to the compound-supplemented medium. The control group, intended to account for any effects of the solvent alone, received treatment with the same solvent used to dissolve the compound. This control group was exposed to the solvent-supplemented medium at an equivalent concentration (0.005%) as in the experimental group. Cell viability was assessed using the 3-(4,5-dimethylthiazol-2-yl)-2,5-diphenyltetrazolium bromide assay (MTT).

**MTT assay**

To assess the cytotoxic effects of DMSO and 2F-PerAcFuc on the KYSE-30 cell line, the MTT assay was performed. KYSE-30 cells were seeded in 96-well plates at a density of 5,000 cells per well. The cells were then treated with different concentrations of DMSO, ranging from 0.001% to 0.5%, and 2F-PerAcFuc dissolved in DMSO, also at various concentrations. Control wells containing cells without any treatment were included. Each experiment was performed in triplicate. Following the designated incubation period of 24 hr, the culture medium was aspirated from the wells. Then, 100 µl of MTT solution (0.5 mg/ml) was added to each well, and the plates were incubated at 37 °C for 3 hr. During this time, viable cells reduced the MTT solution to formazan crystals. After the incubation period, the formazan crystals were solubilized by adding 100 µl of DMSO to each well. The plates were gently shaken for 10 min to ensure complete dissolution of the formazan crystals. The absorbance was measured at 570 nm using a microplate reader. 

***Sphere formation assay***

The sphere formation assay is a widely used technique to evaluate the self-renewal capacity of CSCs *in vitro*. This assay mimics the three-dimensional microenvironment that supports the growth and maintenance of CSCs, allowing for the characterization of their stem-like properties. For the sphere-forming assay, the serum-free medium supplemented with growth factors (DMEM/F12 with 2% B27 supplement, 20 ng of epidermal growth factor, and 20 ng of basic fibroblast growth factor) was used. We seeded 1×10^5^ cells into low-attachment 6-well plates containing the serum-free medium with growth factors and incubated the plates in a humidified incubator at 37 °C with 5% CO_2_. Each condition was tested in triplicate. After 5-7 days, we examined the wells under an inverted microscope, captured images at appropriate magnifications, and compared them using ImageJ software. CSCs typically form floating spherical clusters, referred to as spheres or tumorospheres.

***RNA extraction and gene expression analysis using quantitative real-time PCR***

After collecting spherical cells and tissue samples, they were processed immediately to preserve RNA integrity and minimize degradation. Total RNA was extracted from the collected samples using a commercially available RNA extraction kit (YTA, Tehran, Iran) according to the manufacturer’s instructions. Briefly, the samples were homogenized using vigorous vortexing to ensure thorough disruption of cells and tissues. Following homogenization, the samples were mixed with the RNA extraction reagent (YTzol). The RNA concentration and purity were determined using a spectrophotometer (NanoDrop). A SYBR Green PCR Master Mix was prepared, containing the reverse transcriptase enzyme (M-MLV RT), random hexamer primer, oligo (dT), dNTPs, and RNasein, to make first-strand cDNA from 1000 ng of RNA samples (YTA, Tehran, Iran). In separate wells, the total RNA from each sample was evaluated in triplicate for each target RNA. A Bio-Rad Real-Time QPCR System (United States) was used to perform quantitative real-time reverse transcription PCR (qRT-PCR). The primer sequences used in this research are listed in Table 1.

***Flow cytometry analysis***

The suspended cells were extracted by centrifugation at 1000 rpm for 5 min, followed by trypsin digestion. Negative control, isotype control, and positive samples were separated into three Eppendorf tubes and resuspended in 1 ml of phosphate-buffered saline (PBS). The samples were incubated with 100 µl of a specific primary antibody for 30 min at 4 °C. Then, the samples were centrifuged, the supernatant was discarded, and the pellets were suspended in 1 ml of PBS containing 2% bovine serum albumin (BSA). They were then incubated for 30 min at 4 °C with 200 µl of secondary antibodies at a dilution of 1:100. Next, the pellets were suspended in 500 µl of PBS after being washed twice. The processed samples were evaluated using flow cytometry equipment to detect specific biomarkers.

***Cell invasion assay***

The cell invasion assay (Cultrex BME Cell Invasion Assay) was performed to evaluate cell migration through the extracellular matrix, which is a critical function in biological processes, including cancer cell metastasis. Briefly, 2F-PerAcFuc-treated cells and the control group were grown in serum-free media for 24 hr. The top chamber of the cell invasion device was coated with 50 µl of BME solution. The cells were then harvested and counted at a concentration of 1×10^6^ cells/ml. In each well of the top chamber, 50 µl of cells were placed, and in the bottom chamber, 150 µl of medium containing 5% FBS was added per well. After 24 hr, the bottom chamber was aspirated, and each well was washed twice with wash buffer. After 1 hr, 100 µl of Calcein AM was added to the bottom chamber and incubated. The total number of cells that migrated into the bottom chamber was detected using a fluorescent 96-well plate reader at 485 nm excitation and 520 nm emission.

***Wound-healing assay***

Migration is a vital characteristic of live cells and is essential for cancer metastasis. The wound-healing assay is a method used to assess migration ability. The wound-healing experiment was conducted using KYSE-30 cells (1×10^5^) seeded into six-well cell culture plates and allowed to grow as a monolayer to 70–80% confluence. A sterile one-ml pipette tip was used to lightly scratch the monolayer. Then, 100 µg of 2F-PerAcFuc was added to each well of the 2F-PerAcFuc group, and the cells were cultured for 24 hr. Bright-field microscope (Olympus) images were captured to study the size of the wound area or the distance between wound edges at 0, 1, 2, 6, 12, and 24 hr. The plates were photographed, and the images were subsequently compared using ImageJ software.

***Tumor xenografts***

On day one, a single-cell suspension of KYSE-30 cells (1×10^6^ cells in 100 µl of DMEM/F12) was mixed with Matrigel (BD Biosciences, USA) at a 1:1 volume ratio. The mixture was then injected subcutaneously into the right flank of six-to-eight-week-old male BALB/c nude mice. The mice were randomly assigned to two groups: the control group and the 2F-PerAcFuc-treated group (3 mice/group). Tumor growth was monitored by measuring the tumor dimensions every 3–4 days using calipers. In the 2F-PerAcFuc-treated group, the compound was injected directly into the tumor at a dose of 100 µg on three specific time points: days 7, 14, and 21 after the initial cell injection. The control group received equivalent volumes of the solvent (DMSO) used for compound administration. After twenty-eight days of cell injection, the mice were sacrificed, and tumor tissue was collected and frozen. The tumor volume (V) was calculated using the formula: V = W2 ×L ×0.5, where W and L represent the width and length of the tumor, respectively. All procedures and experiments including, animals were approved and carried out in accordance with the rules of the Animal Ethics Committee of the Golestan University of Medical Sciences (code:ir.goums.rec.1397.018).

***Hematoxylin and eosin (H*** ***&*** ***E) staining***

First, the frozen portions of tissue were placed on a gelatinized surface. Then, the surface was immersed in hematoxylin for 1 min for staining. After a thorough cleaning, it was soaked for 30 sec in a bluing agent, washed in water, and immersed momentarily in 95 percent ethanol. Also, the cytoplasm was immersed in eosin for 1 sec before swiftly dipping in water. Then, it was immersed in 1 change of 95 percent ethanol for 1 min, then 3 changes of 100 percent ethanol for 10 dips each to dehydrate. It was immersed for 2 min in 3 different variations of citrosol. The glass coverslips were used to mount slides.

***Lectin blot assay***

We performed an Aleuria Aurantia lectin (AAL) blot analysis to investigate the expression of fucosylated glycoconjugates in the tumor tissue of nude mice. Protein extracts from the tumor tissue were subjected to gel electrophoresis and transferred onto a nitrocellulose membrane. After blocking, the membrane was probed with Aleuria aurantia lectin (dilution 1:1000) (Vector Labs) following an overnight blocking with PBS containing 3% bovine serum albumin at 4 °C. Subsequently, the membrane was incubated with streptavidin-POD as a secondary antibody (dilution 1:2000) for 1 hr at room temperature, after rinsing with Tris-buffered saline containing 0.05% Tween 20 (TBST, pH 7.4). Then, it was rinsed 3 times with TBST, and Diaminobenzidine (DAB) was added for visualization. As a relative loading control, anti-β-actin was included.

***Immunohistochemistry (IHC)***

Using antibodies against CD15s (BioLegend, San Diego, United States) and E-cadherin (Dako, Glostrup, Denmark), immunohistochemistry (IHC) was performed on frozen sections of KYSE-30 xenograft biopsies. The slides were placed at room temperature for 20 min and then washed with PBS. A PBST (PBS+Tween) solution was used to increase permeability. Endogenous peroxidase and biotin activity were blocked by incubating the tissues with 3% hydrogen peroxide in methanol. The tissues were then blocked for 30 min with 3% horse serum before being probed with primary antibodies for 1 hr at room temperature. HRP-conjugated secondary antibodies were used for 1 hr, and the chromogen used was DAB stain. Hematoxylin (Sigma-Aldrich, UK) was used as a counterstain. Slide photographed with Fluorescence Microscope (Olympus BX51).

## Results

***The typical gene expression of CSCs was detected in spheres***

**Figure 1:**
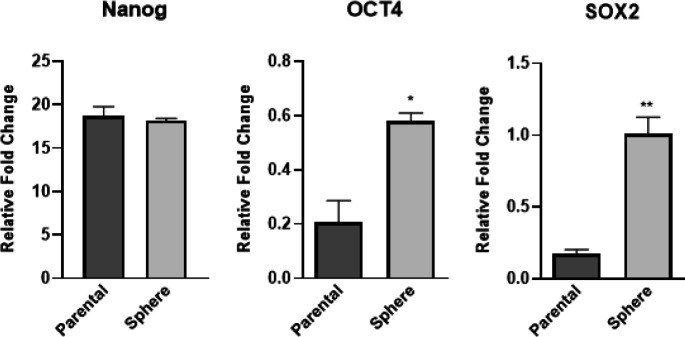
Transcript levels of CSC markers (SOX2, OCT4, and NANOG) in parental cells and spheres

In this study, we evaluated the pluripotency and gene expression of CSC markers. The initial analysis revealed intriguing findings regarding the expression of pluripotent genes, namely Sox2 and Oct4, in relation to the spherical cells derived from KYSE-30 cells compared to the parental cells. Notably, the expression levels of Sox2 and Oct4 were observed to be significantly higher in the spheres, indicating a potential correlation between sphere formation and enhanced pluripotency (Figure 1). These results shed light on the presence of cells within the spheres that possess the remarkable ability to differentiate into various cell types, underscoring their potential for self-renewal. Interestingly, the expression of Nanog, another important pluripotent gene, exhibited similar levels in both the parental cells and the sphere group. This finding suggests that Nanog expression might not be directly influenced by the sphere formation process or the acquisition of pluripotency within these spherical cells. However, it is important to note that Nanog expression alone may not be sufficient to distinguish the cancer stem-like cell properties of the spherical cells. Taken together, these results provide valuable insights into the characteristics of the spherical cells derived from KYSE-30 cells. The higher expression of Sox2 and Oct4, coupled with comparable Nanog expression, signifies the presence of pluripotency and self-renewal capabilities within these cells. These findings contribute to the growing body of evidence supporting the notion that these spherical cells possess key attributes reminiscent of cancer stem-like cells.

***Inhibition of fucosylation decreased fut3 and fut 8 expressions in vitro and in vivo***

**Figure 2:**
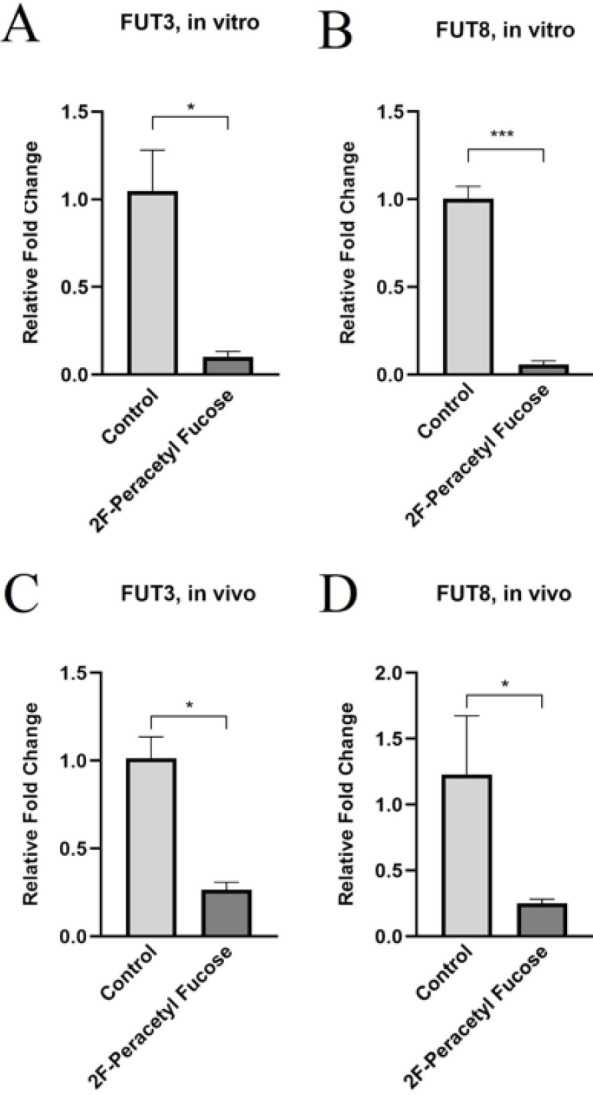
2F-PerAcFuc effects on gene expression

The results of the study demonstrated a significant reduction in the expression levels of fucosyltransferase 3 (FUT3) and fucosyltransferase 8 (FUT8) in both cancer stem-like cells and tumor tissues upon treatment with 2F-PerAcFuc, in comparison to the control groups (Figure 2). The observed decrease in FUT3 and FUT8 expression following treatment with 2F-PerAcFuc suggests the potential of this therapeutic intervention in modulating fucosylation patterns. The down-regulation of these fucosyltransferases could have important implications for cancer biology, as altered fucosylation patterns have been associated with tumor growth and metastasis. These findings highlight the therapeutic potential of targeting fucosylation pathways as a means to disrupt the cancer stem-like cell population and modulate tumor behavior. Further investigations are warranted to elucidate the underlying mechanisms through which 2F-PerAcFuc influences FUT3 and FUT8 expression and to assess its broader impact on cellular functions and tumor progression.

***Inhibition of fucosylation reduced the invasiveness of cancer stem-like cells***

**Figure 3:**
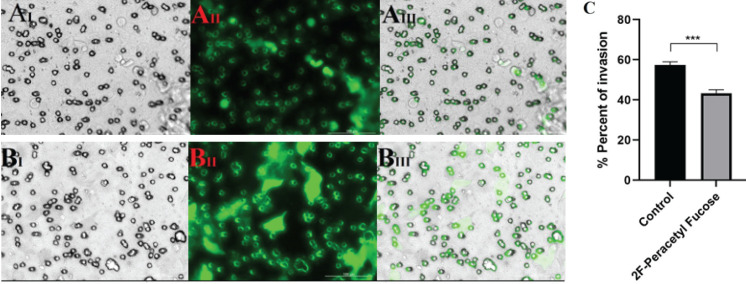
Cell invasion assay results on KYSE-30 cell line

The study findings revealed a noteworthy positive association between the expression level of FUT3 and the invasive potential of cancer stem-like cells. This correlation suggests that FUT3 may play a significant role in promoting the invasion ability of these cells. Moreover, we investigated the impact of 2F-PerAcFuc administration on the invasive behavior of cancer stem-like cells. The results indicated that treatment with 2F-PerAcFuc effectively suppressed the invasion of these cells. To assess cellular infiltration, a transwell assay was conducted using KYSE-30 cells. Remarkably, the results demonstrated a substantial reduction in cellular infiltration into the basement membrane extract (BME) among the cells treated with 2F-PerAcFuc, compared to the control cells (Figure 3). The observed reduction in invasion was statistically significant (*P*<0.01), emphasizing the efficacy of 2F-PerAcFuc in inhibiting cell infiltration in an *in vitro* setting. These findings provide strong evidence supporting the notion that 2F-PerAcFuc exerts a suppressive effect on cell invasion. The investigation of cell invasion using the transwell assay further enhances our understanding of the relationship between 2F-PerAcFuc and the progression of cancer cells. By inhibiting cell infiltration from the basement membrane extract, 2F-PerAcFuc holds promise as a potential therapeutic intervention to impede the invasive behavior of cancer stem-like cells. 

***FUT inhibition resulted in a reduction in the diameter of sphere colonies***

**Figure 4:**
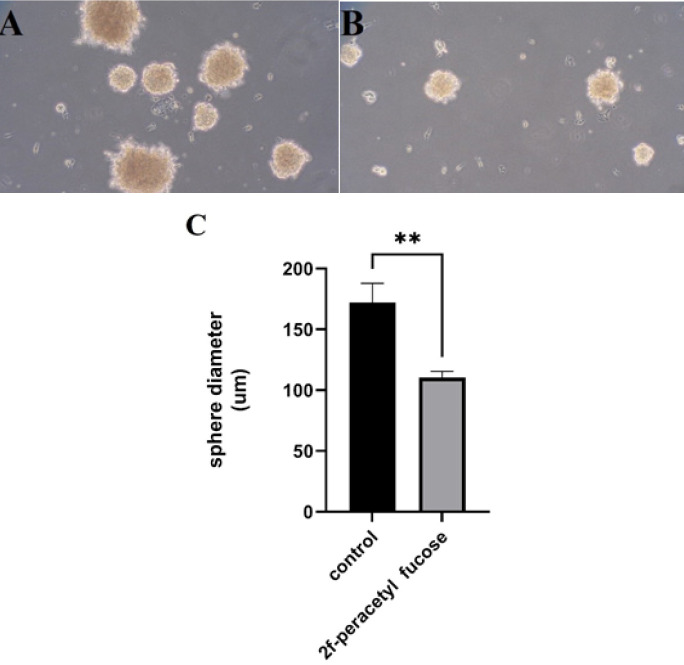
The sphere-forming assay with KYSE-30 cell line

As anticipated, the application of 2F-PerAcFuc demonstrated a significant inhibitory effect on the colonization ability of KYSE-30 cells. The cells treated with 2F-PerAcFuc exhibited a marked reduction in their capacity to form colonies when compared to the control cells. Notably, the colonies formed in the presence of 2F-PerAcFuc were smaller in size compared to the control group (Figure 4). This observation suggests that the treatment with 2F-PerAcFuc not only impedes colony formation but also affects the growth and proliferation of the cells within the colonies. The observed differences in colony phenotype between the treated and control groups could potentially be attributed to the specific cell types involved. Different cell types express distinct growth factor receptors that play crucial roles in regulating cell proliferation. It is plausible that the action of 2F-PerAcFuc may involve the modulation of these growth factor receptors or related signaling pathways, leading to the observed variations in colony size.

***Inhibition of fucosylation by adding 2F-PerAcFuc resulted in decreased CD15s epitope expression in cancer stem-like cells***

**Figure 5:**
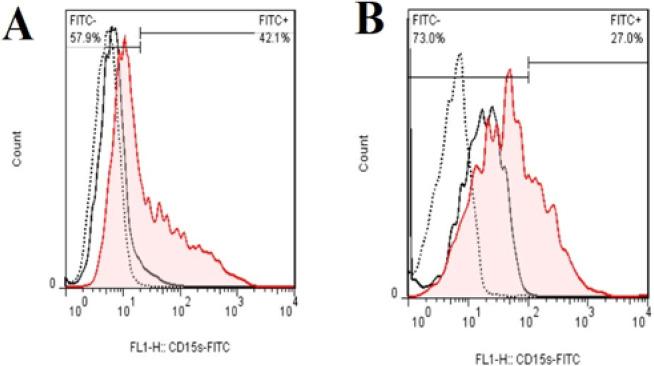
Flow cytometry for the presence of CD15s in cancer stem-like cells

To further assess the impact of 2F-PerAcFuc treatment, flow cytometry analysis was performed to examine the expression levels of CD15s epitope on KYSE-30 cells. The results demonstrated that the cells treated with 2F-PerAcFuc exhibited lower levels of CD15s expression compared to the isotype control, indicating that the treatment effectively reduced the expression of the CD15s epitope on the surface of KYSE-30 cells (Figure 5). These findings provide valuable insights into the role of 2F-PerAcFuc in modulating the expression of CD15s epitope in KYSE-30 cells. The decreased expression of CD15s following 2F-PerAcFuc treatment suggests that the therapeutic intervention can effectively interfere with the fucosylation process mediated by FUT3 and subsequently influence the expression of CD15s epitope.

***Presence of 2F-PerAcFuc led to a reduction in the wound-healing*** ***process***

**Figure 6:**
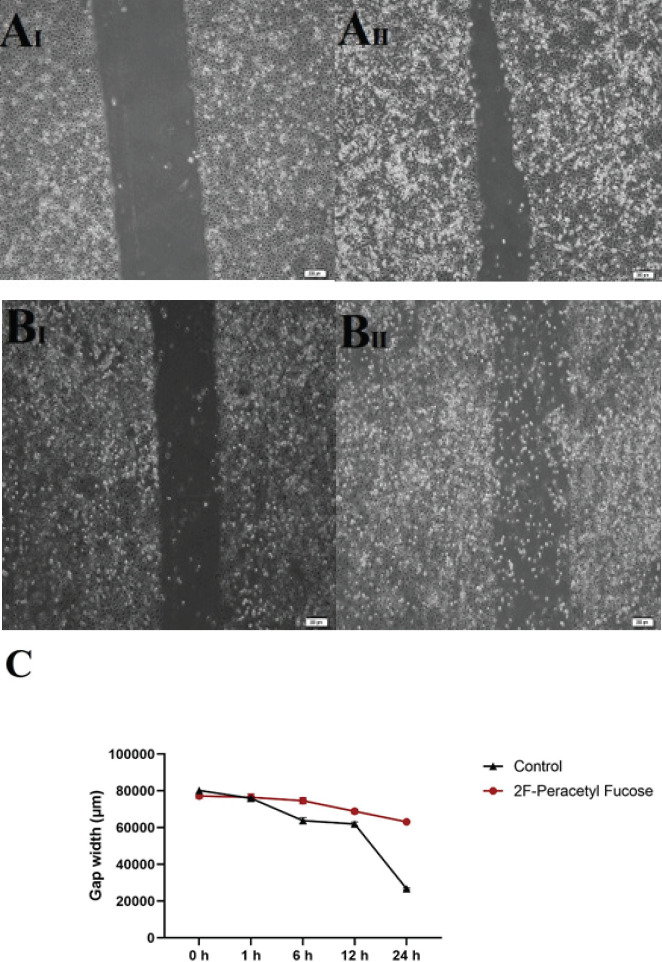
Cell migration was analyzed using a wound-healing scratch assay

Scratch-wound assay was conducted to assess the impact of 2F-PerAcFuc on wound healing. The results of this assay revealed a significant effect of 2F-PerAcFuc on the rate of wound closure when compared to the untreated control group. Specifically, the presence of 2F-PerAcFuc led to a notable decrease in the wound healing rate. After six hours of treatment with a concentration of 100 µg, the wound-healing rate was reduced by approximately 25% compared to the control group (Figure 6). This suggests that 2F-PerAcFuc exerts an inhibitory effect on the migration and closure of wounds in the *in vitro* model. These findings have important implications, indicating that 2F-PerAcFuc may hold therapeutic potential in modulating wound healing processes. By suppressing the wound-healing rate, 2F-PerAcFuc could potentially be utilized in interventions targeting excessive wound healing, such as hypertrophic scars, where controlling the migration and closure of cells is crucial.

***2F-PerAcFuc exhibits non-cytotoxicity in cells***

**Figure 7:**
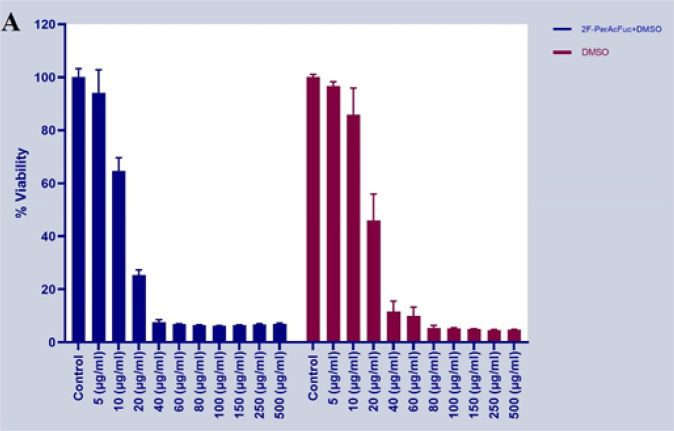
Cytotoxic Effects of DMSO and 2F-PerAcFuc on KYSE-30 cell line

The absorbance values obtained from the MTT assay were used to calculate cell viability as a percentage relative to the control group. The data were expressed as mean ± standard deviation (SD) from three independent experiments. Statistical analysis was performed using [insert statistical software], and significance was determined by [insert statistical test], with *P*<0.05 considered statistically significant. The results demonstrated that DMSO exhibited significant cytotoxicity, while 2F-PerAcFuc did not show any appreciable cytotoxic effects on the cells. Treatment with DMSO at a concentration of more than 0.005% led to a significant decrease in cell viability compared to the control group (*P*<0.05). However, treatment with various concentrations of 2F-PerAcFuc dissolved in DMSO did not result in considerable cytotoxicity on KYSE-30 cells compared to treatment with DMSO alone (Figure 7). 

***Caliper measurements were performed to determine the subcutaneous xenograft tumor volume in nude mice***

**Figure 8:**
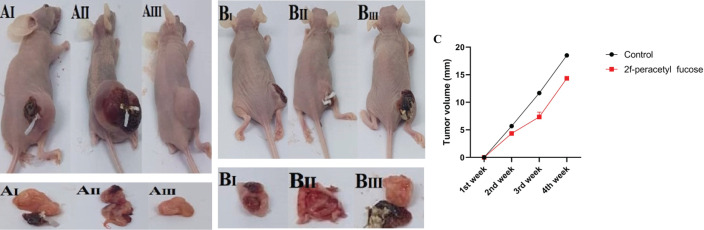
Tumorigenicity in BALB/C nude mice

In the *in vivo* study using nude mice infected with KYSE-30 cells, we investigated the therapeutic potential of 2F-PerAcFuc by directly administering it into the tumor tissue. The results of this study revealed a reduction in tumor volume in the mice treated with 2F-PerAcFuc compared to the control group. Tumor size was monitored and measured weekly using a caliper throughout the study period. Notably, starting approximately 3 weeks after the initiation of treatment, the mice receiving 2F-PerAcFuc exhibited a noticeable decrease in tumor volume compared to the control group (Figure 8). These findings provide compelling evidence that 2F-PerAcFuc effectively inhibited the growth of KYSE-30 cell xenografts in the nude mice model. The observed reduction in tumor volume indicates an effective therapeutic intervention for suppressing tumor growth *in vivo*.

***Relationship between CD15s/FUT3 and E-cadherin/FUT8 in xenograft model of nude mice***

**Figure 9:**
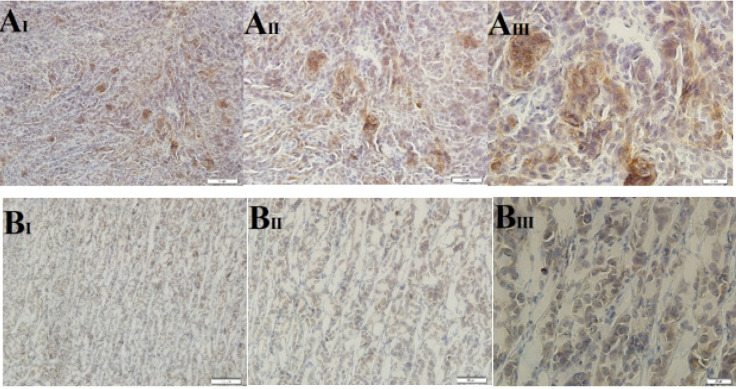
Immunohistochemical staining for CD15s, KYSE-30 xenograft models

**Figure 10:**
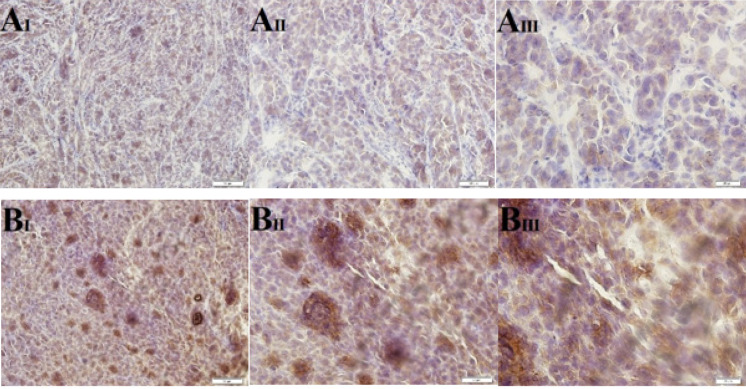
Immunohistochemical staining for E-cadherin, KYSE-30 xenograft models

The immunohistochemical analysis of xenograft tissue samples obtained from nude mice provided valuable insights into the relationship between fucosylation enzymes, cancer cell markers, and tumor behavior. The results indicated that up-regulation of Fut3, an enzyme involved in fucosylation, resulted in an increased expression level of CD15s, a fucosylated marker. However, when 2F-PerAcFuc was introduced, the expression of both Fut3 and CD15s markers decreased, suggesting that the treatment effectively down-regulated fucosylation processes mediated by Fut3 and subsequently impacted the expression of CD15s (Figure 9). In the control group, the expression of E-cadherin, a critical factor involved in cell adhesion, was lower compared to the group treated with 2F-PerAcFuc. Furthermore, the enhancement of Fut8, another fucosylation enzyme, led to increased fucosylation of E-cadherin. As a consequence, the cells became detached from their positions, as illustrated in Figure 10. These findings collectively highlight the impact of fucosylation enzymes on the expression of cancer cell markers, such as CD15s and E-cadherin. Up-regulation of fucosylation enzymes, as observed with Fut3 and Fut8, can lead to alterations in the expression of these markers. Such alterations may create a favorable microenvironment for metastasis, as they affect cell adhesion and promote detachment from the primary tumor site.

***Reduction in the overall amount of fucosylated glycoconjugates in nude mice treated with 2F-PerAcFuc was not significant***

**Figure 11:**
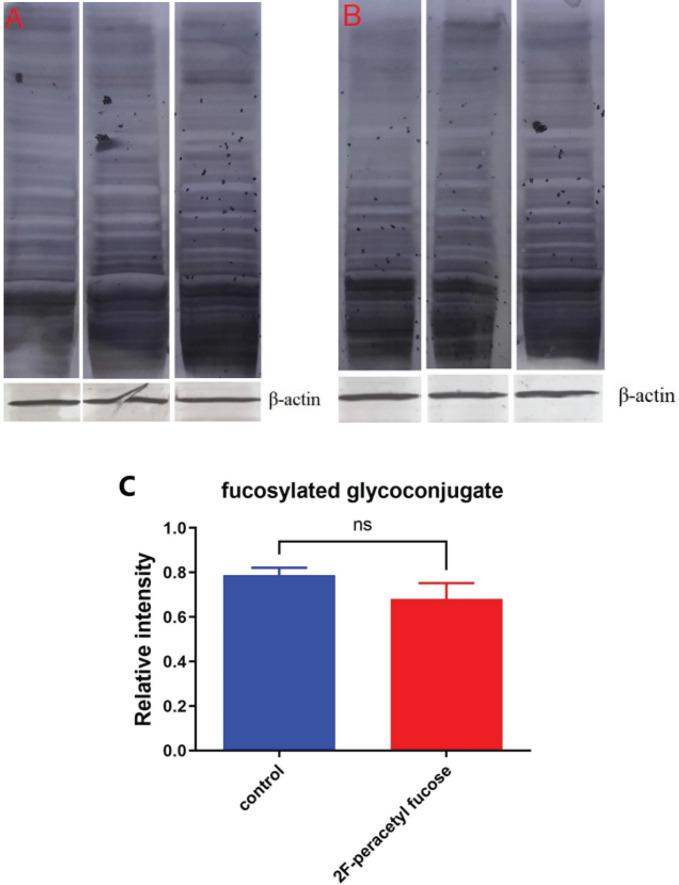
**. ** Immunodetection of fucosylated glycoconjugates by lectin blot using Aleuria Aurantia lectin (AAL)

The AAL blot analysis was conducted to evaluate the relative abundance of fucosylated glycoconjugates in the tumor tissue samples. Upon visual examination of the blot, it was observed that the band intensities varied among the samples. In the control group samples, an intense band was detected, indicating a high expression level of fucosylation. This suggests that fucosylated glycoconjugates were abundant in the tumor tissue of the control group. On the other hand, the 2F-PerAcFuc group samples exhibited a weaker band on the blot, indicating a lower abundance of fucosylation compared to the control group. This suggests that the administration of 2F-PerAcFuc led to a reduction in fucosylation levels in the tumor tissue samples. However, it is important to note that no significant difference in the overall band intensity was observed between the two groups (*P*> 0.05) (Figure 11). This implies that although there were variations in the band intensities, the overall level of fucosylation did not show a statistically significant difference between the control and 2F-PerAcFuc groups. These findings suggest that while the administration of 2F-PerAcFuc may have influenced the abundance of fucosylated glycoconjugates, it did not lead to a significant overall change in fucosylation levels in the tumor tissue samples.

**Table 1:** Sequences of primers used for the real-time PCR reaction on KYSE-30 cell line

Gene	Forward (5ˈ-3ˈ)	Reverse (5ˈ-3ˈ)
Sox2	GATCAGCATGTATCTCCCCG	CCCTCCCATTTCCCTCGTTT
Oct4	GGGCTCTCCCATGCATTCAAAC	CACCTTCCCTCCCAACCAGTTGC
Nanog	GTTTGGGATTGGGAGGCTT	CGATGCAGCAAATACGAGACC
β-actin	AGGCGGACTATGACTTAGTTGCGTTACACC	AAGTCCTCGGCCACATTGTGAACTTTG
Fut3	TCCCTTTTCGTCACACTCAGG	ACCGAACTGGTCTAAGCCTTG
Fut8	TTCAAGTGGTCGAGCTTCCC	GTACAAGTCGATCTGCGAGGT

The expressions of the SOX2 and OCT4 genes were significantly higher in the sphere cells compared to the parental group. However, there were no significant differences observed in NANOG gene expression between the parental and sphere cells.* *P*<0.05, ** *P*<0.01.

Inhibition of FUT3 and FUT8 gene expression was observed upon treatment with 2F-PerAcFuc. The down-regulation of fut3 and fut8 was observed both* in vitro* and *in vivo *compared to the control group. The reported results represent the mean ± standard deviation (SD) of three independent experiments.* *P*<0.05, *** *P*<0.001.

The treatment with 2F-PerAcFuc resulted in a reduction in the invasive potential of cancer stem-like cells. A comparison was made between the control cells (A) and the 2F-PerAcFuc treated cells (B). The cells were stained with Calcein-AM and the assay chamber was read at 485 nm excitation and 520 nm emission. Images of the stained invaded cells were captured using a fluorescent microscope with Bright field (I), GFP filter (II), and merge (III) (Magnification ×100). The bar graph (C) represents the number of cells that invaded the basement membrane. n = 3, ***P<0.0001 for 2F-PerAcFuc treated cells compared to control.

2F-PerAcFuc treatment resulted in a decrease in the colony formation capacity of KYSE-30 cancer cells.The cells were cultured in a serum-free medium supplemented with 2% B27, 20 ng/ml EGF, and 20 ng/ml FGF. In the control group (A) and the group treated with 2F-PerAcFuc (B), cells were cultured for 7 days in 6-well ultra-low attachment dishes, and the diameter of spheres per well was counted. The tumorspheres obtained were observed under a microscope (scale bar: 50 μm). Quantification of the average diameter of KYSE-30 cells in the control and 2F-PerAcFuc groups (C). The tumorsphere diameter is presented as the mean±SD from 3 replicated wells. *P<0.05, **P<0.01.

The flow cytometry analysis of sphere cells treated with 2F-PerAcFuc (sample B) compared to the control group (sample A) revealed a reduction in CD15 expression. This can be observed in the flow cytometry histograms, where the red line represents the staining for CD15s, the black line represents the isotype control antibody, and the black dotted line represents the unstained sample. The shift towards lower fluorescence intensity in sample B indicates a decrease in CD15 expression compared to the control group. This result suggests that 2F-PerAcFuc treatment effectively reduces the expression of CD15s on the surface of sphere cells.

The results of the wound healing scratch assay indicate that the treatment with 2F-PerAcFuc had a negative impact on the closure of the artificially created scratch compared to the control group. The microscopic picture shows that the control group (A) exhibited migration and closure of the scratched area, while the 2F-PerAcFuc treated group (B) showed less migration and the scratch remained open. The bar graph (C) represents the quantification of the filled area, demonstrating reduced closure in the 2F-PerAcFuc treated group compared to the control group. These findings suggest that the concentration of 2F-PerAcFuc negatively affected the wound-healing process in KYSE-30 cells.

The MTT assay was used to compare the cytotoxic effects of DMSO and 2F-PerAcFuc on KYSE-30 cells. Treatment with DMSO at a concentration of more than 0.005% resulted in a significant decrease in cell viability compared to the control group (*P*<0.05). In contrast, treatment with various concentrations of 2F-PerAcFuc dissolved in DMSO did not exhibit significant cytotoxicity on KYSE-30 cells (*P*>0.05). Data are presented as mean±SD from three independent experiments.

2F-PerAcFuc inhibits tumor growth in a nude mouse xenograft tumor model. The cells (1×106) were inoculated subcutaneously into the right side of the back to form a tumor mass. Representative images of mice bearing tumor xenografts for the control (A) and 2F-PerAcFuc (B) groups, as well as their tumors, were shown (n = 3 per group). The tumor volume was assessed each week by calipers and calculated as the length × width × width × 0.5. The tumor growth curve in nude mice is presented (C).

(A) CD15s expression is enriched in the tumor tissue of the control group (61% positive cells), while lower expression (26% positive cells) was observed in tumor tissue samples treated with 2F-PerAcFuc (B). Images were obtained at magnifications of 100× (I), 50× (II), and 20× (III), respectively.

The membranous expression of E-cadherin was 31% positive cells in the control group (A), while the presence of E-cadherin was detected in the central part of the 2F-PerAcFuc-treated cancer tissue was 45% positive cells (B). Images were obtained at magnifications of 100× (I), 50× (II), and 20× (III), respectively.

2F-PerAcFuc treatment led to a reduction in the overall amount of fucosylated glycoconjugates, but this reduction was not statistically significant. Western blot analysis was performed on protein extracts from the xenograft tumor tissue using a biotinylated AAL and streptavidin-POD as the secondary antibody. β-actin was used as a loading control. Representative images of the control group (A) and 2F-PerAcFuc group (B) are shown. The expression levels of fucosylated glycoconjugates are presented as relative intensity (C).

## Discussion

The mechanisms causing altered glycan structures in cancer cells are unknown; however, it is thought that one possible phenomenon is misregulated expression of glycosyltransferases (34, 35). The tissue-specific pattern of glycosyltransferase gene expression, regulation, and its link to the pathogenesis of cancer is still in the early stages of investigation (36-38). Altered glycans can stimulate tumor cell attachment. Cancer-associated glycans, such as SLeX, have been found in almost every type of cancer (39) and interact with selectins to promote tumor cell adherence to endothelial cells, initiating metastasis (40, 41). Conversely, aberrant glycosylation can affect the function of cell adhesion proteins like E-cadherin, a transmembrane glycoprotein, leading to reduced cell-cell adhesion and aiding in the dissociation of cells from the original tumor (42). Previous studies have shown high expression of fut3 and fut8 in tumor-sphere and ESCC tumor tissues compared to matched healthy controls (43). FUT3 is a member of a family of 13 fucosyltransferases that catalyzes the transfer of L-fucose from GDP-fucose to subterminals of GlcNAc of type 1 chain glycolipids and oligosaccharides via an alpha-(1,4) linkage, as well as the subterminal GlcNAc of type 2 chain oligosaccharides via an alpha-(1,3) linkage. FUT8 is the only FUT that catalyzes the alpha-(1,6) activity and conjugates fucose onto the core structure of N-glycans. In our study, we aimed to investigate the metastatic behavior of esophageal cancer stem-like cells after inhibiting the FUT enzymes. In the previous study, we showed that FUT 3 and FUT 8 genes up-regulated in esophageal CSCs (43). Our current experiments revealed marked up-regulation of fut3 and fut8 expression in the kyse-30 cell line, directly regulating the production of SLeX in this cell line. Similar up-regulation of Fut3 expression has been observed in the serum of patients with liver and colorectal cancer (44, 45). Furthermore, higher levels of core fucosylated proteins have been detected in the serum of patients with prostate cancer (46, 47). Overexpression of the Fut3 gene has been implicated in the development of prostate cancer (48). Core fucosylation has been identified as a potential therapeutic target for inflammatory bowel disease, and decreased core fucosylation in CD4+ T cells has shown a protective effect on the course of T cell-mediated colitis (49). We demonstrated that inhibition of fut3 and fut8 using 2F-PerAcFuc directly reduced the production of CD15s and core fucosylation of E-cadherin in the xenograft model of tumor tissue in nude mice. Additionally, according to other studies, FUT enzymes with CD15 expression can disrupt the blood-brain barrier, enabling lung cancer cells to migrate to the brain and form metastases (50]. These findings align with studies suggesting that knocking down FUT genes can potentially inhibit the biosynthesis of certain glycoconjugates on the tumor cell surface (51, 52). Alpha-(1,3/4) and alpha-(1,6) fucose residues can be recognized by AAL (53). AAL blot analysis showed a strong signal for fucosylated glycoconjugates in the control group, indicating a higher level of fucosylation. However, tumor tissues treated with 2F-PerAcFuc in nude mice exhibited reduced fucosylation. Moreover, fucosylation is more common in cancer stem cells compared to parental tumor cells (13), and overexpression of FUT3 is typically associated with cancer progression and poor prognosis (54). Up-regulation of FUT3 enzymes combined with increased SLeX expression has been reported in stem-like cells and associated with increased metastatic properties (55). Prostate cancer cells treated with fucosylation and sialyltransferase inhibitors induce apoptosis and down-regulate the expression of genes and proteins important in cancer progression (56). Consistent with these findings, we demonstrated that fucosyltransferase gene expression is up-regulated in cancer stem-like cells, and this subset of cells exhibits more aggressive properties. On the contrary, other investigations have revealed that fucosylation activates death receptor 5 in colon cancer cells (57), and loss of a1,6-fucosyltransferase inhibits liver regeneration, while L-fucose, which can enhance GDP-fucose production through a salvage pathway, significantly promotes the delayed liver regeneration (58). In lung cancer cells, sialylation and fucosylation of the epidermal growth factor receptor inhibit dimerization and activation (59), and decreased core fucosylation has been implicated in gastric cancer carcinogenesis (60). Considering the tissue-specific expression pattern of fucosyltransferase enzymes, it is conceivable that inhibition of fucosylation could reduce the metastatic behavior of cancer cells with high expression levels of fucosyltransferases, such as the esophageal cancer cell line kyse-30. 

## Conclusion

Based on our data, it is evident that inhibition of FUT3 and FUT8 plays a significant role in the production and stability of CD15s and E-cadherin, which are important cancer cell markers. Remarkably, our findings highlight their involvement in promoting the metastatic capacity of cancer stem cells in esophageal cancer. By blocking the over-activity of FUT3 and FUT8 and subsequently inhibiting the expression of the CD15s epitope while stabilizing E-cadherin contact inhibition, it may be possible to impede the metastatic potential of esophageal cancer cells. Further research and development of targeted therapies aimed at these specific markers could potentially improve the clinical outcomes for patients with esophageal cancer.

## Authors’ Contributions

A RA conceived and designed the study, and contributed to data collection and analysis and manuscript writing. J A supervised the study, provided critical input in study design, and revised the manuscript. A K provided expertise in study design, gave critical feedback on the manuscript, and assisted in manuscript writing and revision. A G provided expertise in study design, gave critical feedback on the manuscript, and assisted in manuscript writing and revision.

## Conflicts of Interest

The authors declare no competing interests.
